# Target enrichment using parallel nanoliter quantitative PCR amplification

**DOI:** 10.1186/1471-2164-15-184

**Published:** 2014-03-10

**Authors:** Bram De Wilde, Steve Lefever, Wes Dong, Jude Dunne, Syed Husain, Stefaan Derveaux, Jan Hellemans, Jo Vandesompele

**Affiliations:** 1 Center of Medical Genetics Ghent, Ghent University, Ghent, Belgium; 2 WaferGen Biosystems Inc, Fremont, USA; 3 WaferGen Biosystems Europe S.à r.l, Luxembourg, Luxembourg; 4 Biogazelle, Zwijnaarde, Belgium

**Keywords:** Next generation sequencing, Target enrichment, Sequence capture, Quantitative PCR, NCI60, Mutation detection

## Abstract

**Background:**

Next generation targeted resequencing is replacing Sanger sequencing at high pace in routine genetic diagnosis. The need for well validated, high quality enrichment platforms to complement the bench-top next generation sequencing devices is high.

**Results:**

We used the WaferGen Smartchip platform to perform highly parallelized PCR based target enrichment for a set of known cancer genes in a well characterized set of cancer cell lines from the NCI60 panel. Optimization of PCR assay design and cycling conditions resulted in a high enrichment efficiency. We provide proof of a high mutation rediscovery rate and have included technical replicates to enable SNP calling validation demonstrating the high reproducibility of our enrichment platform.

**Conclusions:**

Here we present our custom developed quantitative PCR based target enrichment platform. Using highly parallel nanoliter singleplex PCR reactions makes this a flexible and efficient platform. The high mutation validation rate shows this platform’s promise as a targeted resequencing method for multi-gene routine sequencing diagnostics.

## Background

The advent of next generation sequencing technology has unleashed a wealth of targeted resequencing experiments in all fields of genomics [1]. The field of multi-gene disease diagnostic sequencing is changing rapidly with a shift from conventional Sanger sequencing to targeted next generation sequencing. In addition, many researchers face the daunting task of validating large sets of genomic variants resulting form large scale resequencing studies that investigate the human exome or whole genome [2-5]. Sanger sequencing has long been the gold standard sequencing technology and remains an important method for small scale sequencing experiments and routine genetic diagnostics. Compared to next generation sequencing, Sanger sequencing is a labor intensive and relatively expensive technology. Both the PCR sequencing reaction and interpretation of the sequencing trace files are a time consuming processes making Sanger not the most ideal technology for multi-gene studies or large scale variant confirmation. Even for a diagnostic target for which validated sequencing assays are available, interpretation of the Sanger trace file is a semi-automatic process at best, often requiring human review (see Mitchelson et al. for review) [6,7]. More so, in many genetic studies sample heterogeneity or exceptions to the classical bi-allelic state of the genome make this analysis even more challenging, if not impossible.

Next generation sequencing can tackle most of these challenges. The release of bench-top scale sequencing machines has paved the way to multi-gene targeted next generation sequencing diagnostics. The challenge of the upfront target enrichment has now become the bottleneck for many sequencing applications. Many probe or PCR based single tube sequence capture techniques currently exist. These methods typically require extensive optimization to reach the quality standards set in many Sanger sequencing diagnostic facilities. Most diagnostic labs have already invested in the optimization of PCR assays for the genomic regions of interest; it is therefore problematic to perform this optimization again in order to switch sequencing platforms.

Here, we present a new platform for detecting genetic variants directed at multi-gene disease diagnostics. By optimizing several steps in a custom PCR based sequence enrichment strategy and upscaling this strategy using a highly parallel nanoliter quantitative PCR instrument, we developed a highly flexible enrichment protocol that has a high efficiency, a near perfect target specificity and scales to address the challenges discussed earlier. Our workflow allows the selective resequencing of hundreds to a few thousands of targets in a single analysis, greatly reducing the overall validation cost and effort. It even allows the researcher to re-use previously optimized assays in a highly parallel fashion. In a proof of concept study, we have rediscovered known mutations in well characterized cancer cell lines. In addition, we have used objective quality parameters that enable transparent and robust inter-platform comparisons.

## Methods

For this technical proof of concept study we aimed at resequencing a set of genes known to be mutated in cancer samples. We selected 15 cell lines from the NCI60 panel [8] for which high quality mutation data are made available through the cosmic database [9] for a large list of known cancer genes.

### Samples

A selection of 15 cancer cell lines and 2 normal control samples were included in this study (Table 1). In addition, the enrichment was repeated on the two normal control samples and one of the cell lines (MCF7) to evaluate the technical reproducibility of the platform. A total of 360 pg of input DNA (~112 gene copies) was used per nanoliter PCR reaction.

**Table 1:** Samples and known mutations

**Samples**		**Mutations:**			
**Name**	**Type**	**Gene name**	**Genomic location**	**cDNA position**	**Protein position**
BT-549	Breast cancer cell line	PTEN	10:89720672	c.823delG	p.V275fs*1
CCRF-CEM	Leukemia cancer cell ine	CDKN2A	9:21968228	c.1_471del471	
			9:21971002	c.317_522del206	
		MLH1	3:37042536	c.298C > T	p.R100*
			3:37056036	c.790 + 1G > A	
		NOTCH1	9:139399362	c.4783_4784ins36	p.R1595 > PRLPHNSSFHFLR
		PTEN	10:89653782	c.80_492del413	
HCT116	Colon cancer cell line	CDKN2A	9:21974758	c.68_69insG	p.R24fs*20
			9:21994234	c.220_220delG	p.E74fs*15
		MLH1	3:37056000	c.755C > A	p.S252*
IGROV-1	Ovarian cancer cell ine	MLH1	3:37070378	c.1513delA	p.S505fs*3
		MSH6	2:48030647	c.3261delC	p.F1088fs*2
PC3	Prostate cancer cell line	PTEN	10:89685270	c.165_1212del1048	p.R55fs*1
SN12C	Kidney cancer cell line	NF2	22:30032739	c.115-1G > C	
786-O	Kidney cancer cell line	CDKN2A	9:21974677	c.1_150del150	
			9:21984138-21984453	c.1_316del316	
		PTEN	10:89692961	c.445C > T	p.Q149*
		VHL	3:10183842	c.311delG	p.G104fs*55
ACHN	Kidney cancer cell line	CDKN2A	9:21968228	c.1_471del471	
			9:21971002	c.317_522del206	
		NF2	22:30032794	c.169C > T	p.R57*
DU-145	Prostate cancer cell line	CDKN2A	9:21971108	c.250G > T	p.D84Y
		MLH1	3:37038108	c.117-2A > T	
HCT-15	Colon cancer cell line	APC	5:112177787	c.6496C > T	p.R2166*
			5:112175539	c.4248delC	p.I1417fs*2
		BRCA2	13:32913837	c.5351delA	p.N1784fs*7
			13:32913837	c.3599_3600delGT	p.C1200fs*1
		MSH6	2:48032121	c.3511_3516 > T	p.D1171fs*4
			2:48025990	c.868delC	p.L290fs*1
HT-29	Colon cancer cell line	APC	5:112173848	c.2557G > T	p.E853*
			5:112175957	c.4666_4667insA	p.T1556fs*3
MCF7	Breast cancer cell line	CDKN2A	9:21971002	c.317_522del206	
			9:21968228	c.1_471del471	
MDA-MB-231	Breast cancer cell line	CDKN2A	9:21967751	c.1_522del522	
			9:21968228	c.1_471del471	
		NF2	22:30057209	c.691G > T	p.E231*
MOLT-4	Leukemia cancer cell line	CDKN2A	9:21967751	c.1_522del522	
			9:21968228	c.1_471del471	
		NOTCH1	9:139390649	c.7544_7545delCT	p.P2515fs*4
		PTEN	10:89717775.	c.800delA	p.K267fs*9
OVCAR-5	Ovarian cancer cell line	CDKN2A	9:21967751	c.1_522del522	
			9:21968228	c.1_471del471	
Normal1	Healthy normal1 control DNA				
Normal2	Healthy normal2 control DNA				

NCI60 cell lines and normal control DNA samples included in this study with a list of known mutations per cel line.

In nonsense or frameshift mutations the *symbol indicates the loss of the amino acid coding potential of the DNA sequence.

### Enrichment platform

**Figure 1:**
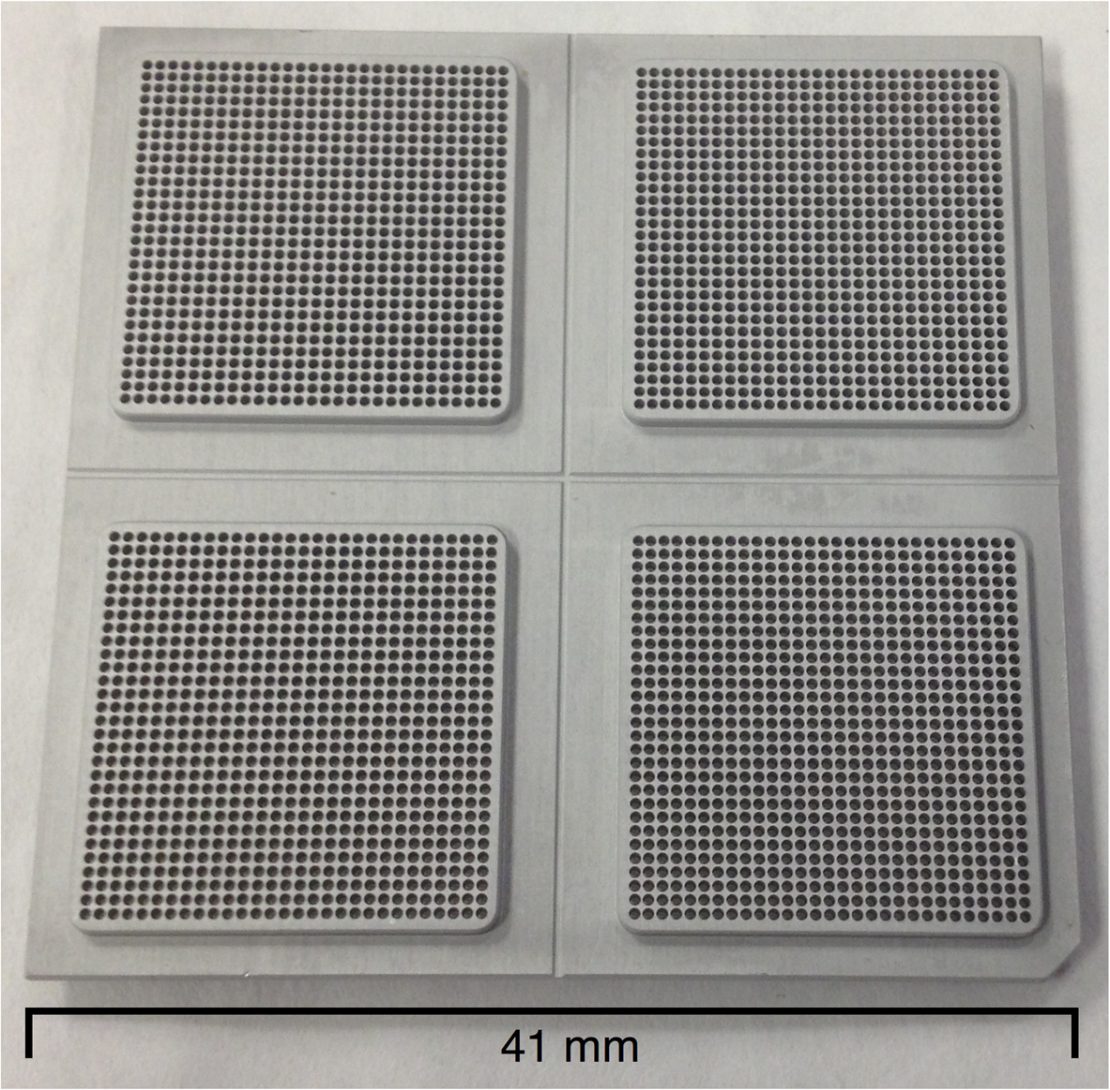
**Nanowell chip.** 4 quadrant nanowell chip used in this experiment.

The SmartChip nanowell platform (WaferGen Biosystems) is an ultra-high throughput quantitative PCR (qPCR) platform used for large scale gene expression studies or digital PCR. To address the problem of PCR product collection, WaferGen Biosystems specifically developed a capture system for the nanowell SmartChip. By reverse centrifuging of the capture chips in custom capture devices, PCR products were collected from the nanowell chips. A prototype capture device for the 5184 reaction well chips has been used in some preliminary testing, but for this study we have used a 4 quadrant chip layout (841 wells per quadrant, see Figure 1) and the matching disposable extraction fixtures to perform target capture of up to 4 samples on a single chip. Evidently, any combination of samples and amplicons is possible; ranging from 4 times a maximum of 841 amplicons, 2 times up to 1682 amplicons or 1 time 3364 amplicons, allowing for a maximum in experimental flexibility. The MyDesign dispenser provides exact control over the dispensing of primers and samples in the reaction wells.

### Target enrichment

A list of 16 known cancer genes with diagnostic relevance was selected for resequencing (Table 2). The genes were selected to harbor mutations in the selected NCI60 cell lines. Primers were designed to amplify all exons of the genes using primerXL, our quantitative PCR primer design tool adapted for resequencing primer design (http://www.primerxl.org) (Lefever et al., in preparation). A total of 376 amplicons were designed using tiling settings, taking into account known SNP positions and with a target annealing temperature of 60°C. The average amplicon length is 441 basepairs (bp) with a range of 319 to 745 bp. Primer and amplicon information is listed in Additional file 1. The total target region comprised of 165 811 bp. Primers were ordered from Integrated DNA Technologies.

**Table 2:** Genes and target regions

**Name**	**Ensembl ID**	**Number of exons**	**Target region**	**Number of primers**	**Capture size (bp)**
BRCA1	ENSG00000012048	12	1693	33	14202
MLH1	ENSG00000076242	16	6025	18	7416
PALB2	ENSG00000083093	9	9027	19	8072
MSH2	ENSG00000095002	8	4605	20	8095
TGFBR1	ENSG00000106799	63	13147	25	11159
PHOX2B	ENSG00000109132	34	9371	4	2028
MSH6	ENSG00000116062	27	10930	16	7441
VHL	ENSG00000134086	16	10701	4	1896
APC	ENSG00000134982	3	3737	35	15085
BRCA2	ENSG00000139618	10	4328	43	20068
NOTCH1	ENSG00000148400	3	538	32	16514
ATM	ENSG00000149311	3	565	63	26598
TGFBR2	ENSG00000163513	16	3307	19	7987
PTEN	ENSG00000171862	13	4003	10	4329
NF2	ENSG00000186575	4	342	17	6972
FLCN	ENSG00000264187	12	1495	13	5586
Total		249	83814	371	163448

List of the genes targeted, their Ensembl gene id, number of exons and target size.

Dispensing of the reaction components in the SmartChip reaction wells is a 2 step process performed on the SmartChip Multiplesample Nanodispenser. The first step is the dispensing of 50 nl of a primer-combination. For this a 500 nM primer and 2X Bio-Rad SsoAdvanced SYBR PCR mastermix solution is presented in a 384 well plate format to the Nanodispeser machine and dispensed on the SmartChip wells. The second step consists of the dispensing of another 50 nl of a 7.2 ng/μl sample DNA solution to the nanowells containing the primer mastermix combination. The final reaction volume of 100 nl consists of 360 pg of template DNA and 250 nM of forward and reverse primer in a 1X mastermix solution. A rough visual quality control of the dispensing steps is carried out by visualizing the nanowell chips by means of a magnifying glass.

With 376 targets in this experiment we were able to use the 4 quadrant nanowell chips to capture 4 samples per chip. The primers were spotted in duplicate for most of the samples. Some samples were repeated with singlet PCR reaction per chip to evaluate both amplification and DNA extraction efficiency and reproducibility; unused wells were left empty (see Table 3 for experimental replicate configuration).

**Table 3:** Experimental duplicate layout, read and coverage statistics

**Sample**	**Number of PCR replicates**	**Total number of reads**	**Mapped**	**On target**	**PCR duplicates**	**Amplicon**		**Exon**	
						**Mean coverage**	**Standard deviation**	**Mean coverage**	**Standard deviation**
Normal1-duplicate-B	2	951169	97.7%	79.0%	15.6%	542.7	334.9	427.9	409.4
BT-549	2	1031471	97.4%	77.9%	16.6%	567.7	365.2	451.1	436.8
CCRF-CEM	2	894547	97.7%	76.4%	15.2%	498.4	356.9	403.3	413.9
HCT-15	2	826606	97.7%	80.3%	14.0%	486.5	327.9	384.1	387.4
MOLT-4	2	1271883	97.9%	77.3%	17.2%	747.0	460.3	589.9	561.2
HCT116	2	1384151	97.7%	72.0%	21.0%	724.4	524.5	589.1	609.5
Normal1-duplicate-A	2	1608833	97.9%	74.3%	20.6%	877.3	536.2	692.3	658.2
SN12C	2	472237	97.8%	84.7%	9.8%	294.1	199.9	233.5	233.6
IGROV-1	2	672337	97.8%	79.7%	11.6%	404.9	281.6	325.8	329.2
DU-145	2	723007	97.8%	82.1%	12.3%	434.7	273.1	346.9	332.1
MDA-MB-231	2	769668	97.7%	81.2%	13.2%	461.8	315.7	367.6	373.4
OVCAR-5	2	659926	97.3%	81.1%	13.1%	386.5	293.4	308.1	332.7
786-O	2	1167234	97.9%	79.1%	17.2%	684.6	463.9	543.9	549.2
MCF7-B	2	1053960	97.8%	77.8%	17.3%	588.1	398.9	472.1	473.4
PC3	2	566416	97.8%	79.9%	11.5%	329.9	252.1	267.3	287.9
MCF7-A	2	1299077	97.6%	76.0%	19.3%	703.5	465.2	569.0	558.0
RPMI-8226	1	1037872	97.5%	77.2%	16.9%	575.4	394.6	462.1	461.8
Normal2-singleton-A	1	806612	97.7%	79.9%	14.5%	461.2	296.8	367.2	355.5
MCF7	1	1193431	97.6%	75.3%	18.2%	647.8	441.9	515.4	513.5
Normal1-singleton-A	1	1227019	97.9%	74.7%	17.3%	688.5	450.0	543.0	533.9
Normal2-singelton-B	1	571558	97.7%	82.8%	11.3%	340.9	227.4	274.5	268.0
HT-29	1	578229	97.8%	81.7%	11.6%	340.1	243.2	273.1	280.7
Normal1-singleton-B	1	1070055	97.8%	75.9%	15.6%	615.2	393.4	488.2	472.0
ACHN	1	853092	97.8%	79.2%	14.9%	486.6	327.2	387.2	384.2
									
**Mean**		945432.9	97.7%	78.6%	15.2%	537.0	359.3	428.4	425.6
**Stadard deviation**		296401.9	0.1%	3.0%	3.0%	153.0	94.2	121.2	115.0

The number of PCR replicates performed on the capture chip for each target per sample. The number of reads obtained during the sequencing and the coverage this resulted in.

SmartChips containing the assay and samples were cycled in the SmartChip Cycler (WaferGen Biosystems) using the following thermal parameters: 3 minutes at 95°C, 40 cycles composed of 30 seconds at 95°C and 60 seconds at 60°C. This amplification protocol was optimized for sequence enrichment of long PCR fragments and is deviant from the default qPCR protocol in that it has a significantly longer annealing/extension phase. Immediately following amplification, melt curve analysis was performed from 60°C to 97°C (0.4°C/step). After cycling, 4 disposable extraction fixtures were attached to each SmartChip, one fixture per quadrant, and PCR products were collected in 0.2 ml PCR tubes by means of centrifugation (1 sample per tube) at 3500 rpm for 15 minutes.

### Library preparation and sequencing

PCR pools were purified using AMPure beads XT (Beckman Coulter). The concentration of each pool was measured using the dsDNA assay kit on the Qubit fluorometer (Invitrogen) and fragment analysis occurred on a BioAnalyzer 2100 using the high sensitivity chip (Agilent). Library preparation and sequencing was carried out by the Nucleomics core facility at the Flemisch institute for biotechnology (VIB). Nextera XT (Illumina) library preparation on all 24 PCR pools occurred following the manufacturers recommendation using 1 ng of each sample pool as input. In short the Nextera transposase ensures random fragmentation and adaptor ligation to the amplicons after which a dual barcoding occurred during amplification, followed by purification on AMPure beads. The molarity of each library was determined using the concentration (measured by Qubit) and fragment length (BioAnalyzer). Libraries were diluted to equal molarity and finally pooled by using equal volumes of each library. This sequencing pool was diluted to 10 nM, and finally 95% of sequencing pool (6 pM) and 5% of Phix control (8 pM) were mixed and loaded into the flowcell of a MiSeq (Illumina) instrument. Sequencing was performed for 150 bp in paired end mode.

### Data analysis

Raw sequencing data was demultiplexed on the MiSeq instrument using the manufacturer’s software. Of the 23 076 775 reads obtained, 1.6% were lost due to an unrecognizable index. The sequencing resulted in an average read count per sample of 945 432 (range 472 237–1 608 833, see Table 3 for reads per sample). Mapping was performed to build 37 of the human reference genome (Genome reference consortium GRCh37) using BWA [10] (v. 0.5.9). Reads were quality recalibrated using the Genome analysis toolkit [11] (v. 1.6-13-g91f02df) and duplicate reads were removed using Picard tools (v. 1.59).

Variants were called using the Genome Analysis Toolkit unified genotyper [11] (v 1.6-13-g91f02df). Variants were annotated and sample calls were compared using our custom cloud based analysis platform seqplorer (http://www.seqplorer.org) (De Wilde et al., in preparation).

Coverage data was extracted for each sample using samtools depth option on each genomic position. Both the amplicon locations and the coding exon locations of genes captured in this experiment were used as the target locations for calculating the coverage statistics as indicated in the results section. As our goal was to evaluate the capture platform and not the subsequent library prep and sample pooling we eliminated the inter-sample coverage by normalizing the coverage for each position by the mean coverage per sample.

Further statistical analysis and plotting of coverage data was done using the R language and environment for statistical computing (R version 2.15.1; http://www.R-project.org).

## Results and discussion

To evaluate the overall technical performance, efficiency and reproducibility of this novel PCR based sequence enrichment platform we included several levels of quality control. First, through the resequencing of known cancer genes on a set of cancer cell lines with known mutation status, we were able to evaluate the rediscovery rate. Second, SNP calls were compared to public datasets to assess accuracy or compared between replicated samples to evaluate the reproducibility of the variant calling. Third, an objective coverage evaluation among technical replicates as well as different samples was performed.

### qPCR performance metrics

One of the advantages of using a massively parallel quantitative PCR platform for target capture is the upfront quality check that can be performed on the amplification curves. All chip amplification curve profiles looked satisfactory with only 1.16% reaction dropout (defined as a Cq > 29). Although a weak correlation exists between the Cq value of the amplification reaction and the amplicon coverage (R2 = 0.216), no clear correlation was observed between either amplicon length or end-point fluorescence and sequence coverage. Overlapping or tiling assays were excluded from this analysis as the coverage cannot be unambiguously attributed to one of the overlapping assays.

### Coverage analysis

**Figure 2:**
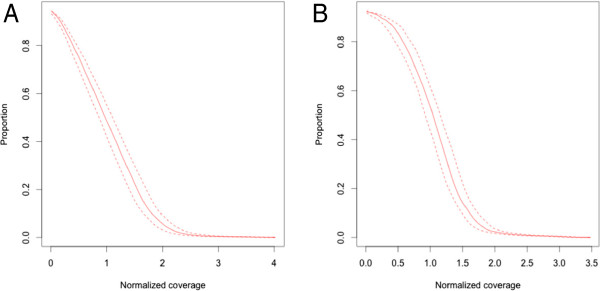
**Mean normalized coverage distribution.** Cumulative distribution plot of mean normalized coverage for the capture amplicons **(A)** and the exons of the genes targeted **(B)**.

We obtained roughly 0.95 (stdev 0.3) million reads per sample. Almost all the reads were mappable to the reference genome and 78.6% (stdev 3%) mapped back to the target region. On average, 15.2% (stdev 3%) of PCR duplicates were present. The success rate in the assay design and target capture was 93.6% as defined by the fraction of assays with a mean coverage > 20-fold in more than 80% of the samples. 96.4% of the assays resulted in coverage in at least one of the samples. As the coverage is a function of the number of reads obtained for a given sample (see Table 3 for detailed per sample read and coverage statistics), data were normalized by dividing the coverage of each base by the mean coverage for that sample. In this way, we can compare the coverage statistics over the samples and evaluate the variation in coverage attributable to the capture platform. Figure 2A and B show the normalized coverage over the amplicons and targeted exons, respectively, and demonstrate that the uniformity and reproducibility of the capture platform is extremely good. 88.7% of the exonic bases receive a coverage between 0.2 and 5 times the mean coverage and 78.1% of the exonic bases fall within a two fold coverage range around the mean.

**Figure 3:**
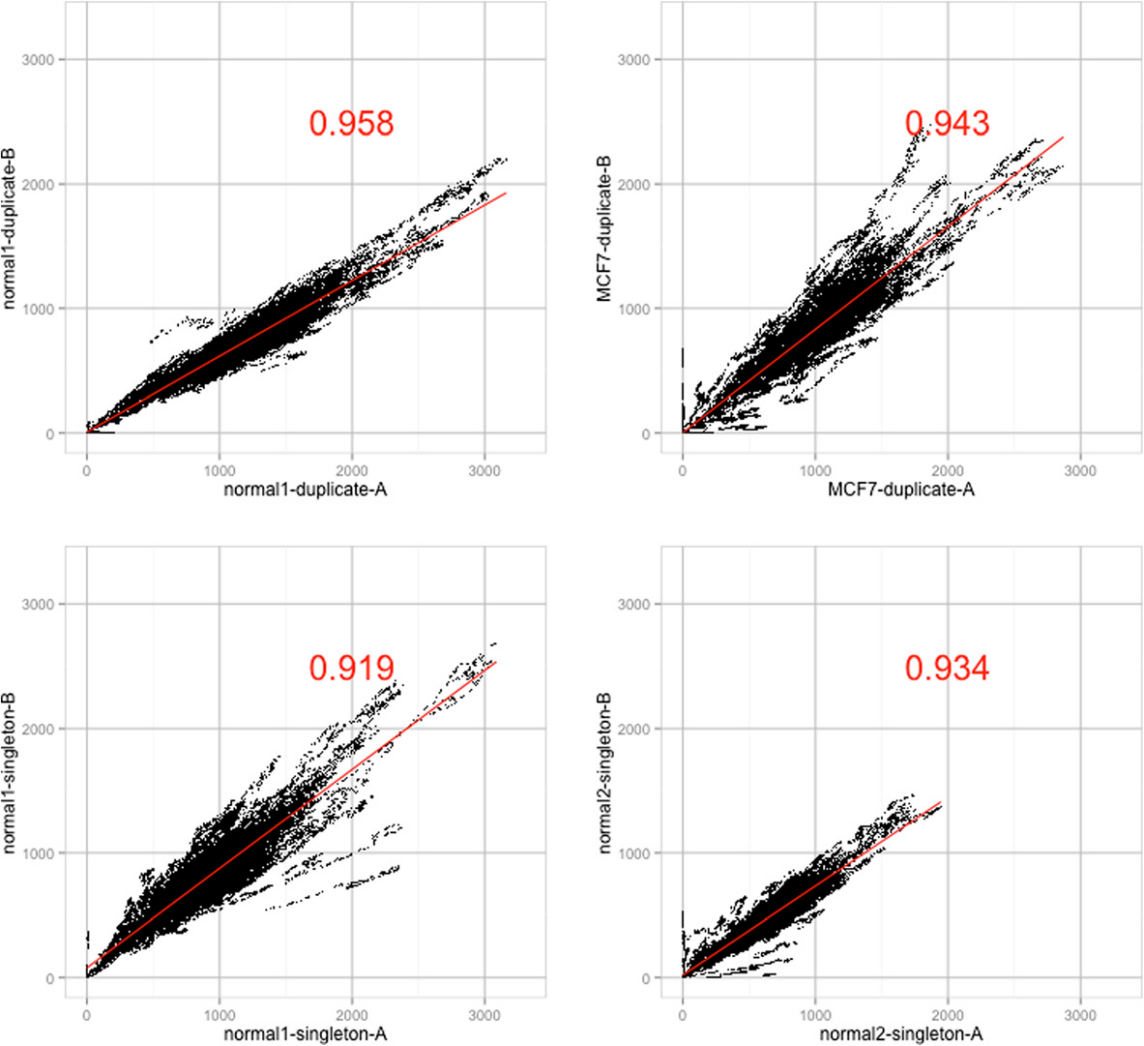
**Technical replicate coverage correlation.** Per base coverage correlation plot and spearman rank correlation values (red) for technical capture replicates.

The high reproducibility of the target capture across the samples is demonstrated by the high average Spearman rank correlation of 0.892 (standard deviation of 0.038) between the coverage values for any two different samples (see Table 4). Figure 3 shows the correlation between the technical replicates. No difference in coverage correlation is apparent between the duplicate and singleton PCR capture reactions.

**Table 4:** Coverage correlation

	**PC3**	**MCF7GhentB**	**ACHN**	**786-O**	**Normal1-singleton-B**	**OVCAR-5**	**MDA-MB-231**	**DU-145**	**IGROV-1**	**SN12C**	**Normal1-duplicate-A**	**HT-29**	**HCT116**	**MOLT-4**	**HCT-15**	**Normal2-singleton-B**	**CCRF-CEM**	**Normal1-singleton-A**	**BT-549**	**MCF7**	**Normal2-singleton-A**	**RPMI-8226**
**MCF7GhentB**	0.884																					
**ACHN**	0.872	0.910																				
**786-O**	0.844	0.864	0.865																			
**Normal1-singleton-B**	0.843	0.901	0.932	0.870																		
**OVCAR-5**	0.798	0.799	0.825	0.904	0.804																	
**MDA-MB-231**	0.846	0.861	0.864	0.965	0.861	0.936																
**DU-145**	0.877	0.909	0.919	0.928	0.920	0.887	0.940															
**IGROV-1**	0.926	0.905	0.900	0.892	0.887	0.798	0.865	0.910														
**SN12C**	0.882	0.901	0.914	0.910	0.902	0.928	0.935	0.953	0.894													
**Normal1-duplicate-A**	0.840	0.945	0.902	0.885	0.915	0.794	0.863	0.916	0.890	0.886												
**HT-29**	0.851	0.888	0.939	0.832	0.924	0.812	0.855	0.890	0.869	0.896	0.868											
**HCT116**	0.946	0.911	0.907	0.887	0.880	0.816	0.875	0.911	0.975	0.906	0.881	0.877										
**MOLT-4**	0.858	0.904	0.900	0.938	0.919	0.853	0.928	0.965	0.917	0.933	0.933	0.864	0.910									
**HCT-15**	0.833	0.852	0.867	0.969	0.858	0.927	0.975	0.938	0.870	0.919	0.868	0.838	0.872	0.931								
**Normal2-singleton-B**	0.842	0.883	0.931	0.832	0.915	0.836	0.854	0.903	0.856	0.906	0.869	0.933	0.867	0.875	0.844							
**CCRF-CEM**	0.959	0.907	0.909	0.870	0.876	0.817	0.865	0.907	0.948	0.902	0.868	0.880	0.960	0.891	0.863	0.881						
**Normal1-singleton-A**	0.832	0.897	0.932	0.864	0.950	0.805	0.859	0.908	0.883	0.901	0.910	0.925	0.879	0.919	0.855	0.913	0.871					
**BT-549**	0.859	0.887	0.903	0.900	0.894	0.933	0.928	0.950	0.867	0.963	0.881	0.891	0.883	0.917	0.913	0.912	0.889	0.889				
**MCF7**	0.840	0.878	0.945	0.827	0.918	0.857	0.855	0.896	0.846	0.919	0.863	0.948	0.865	0.861	0.836	0.942	0.871	0.922	0.928			
**Normal2-singleton-A**	0.861	0.908	0.957	0.855	0.944	0.826	0.865	0.923	0.885	0.913	0.901	0.941	0.892	0.899	0.862	0.946	0.898	0.936	0.913	0.946		
**RPMI-8226**	0.855	0.876	0.945	0.824	0.912	0.838	0.853	0.893	0.846	0.899	0.858	0.950	0.870	0.852	0.839	0.945	0.882	0.910	0.915	0.966	0.956	
**Normal1-duplicate-B**	0.860	0.951	0.917	0.884	0.918	0.829	0.879	0.931	0.889	0.914	0.973	0.893	0.890	0.924	0.880	0.900	0.890	0.914	0.910	0.897	0.922	0.889
**Mean**	0.892																					
**Stdev**	0.038																					

Spearman rank correlation of the per base coverage in between all pairs of individual captures reactions.

### Mutation rediscovery

The NCI-60 cell lines analyzed in this study contain in total 25 well documented mutations [12] in the targeted genes from our resequencing experiment (Cosmic database, December 2012). Two of the variants in the PTEN gene, in samples PC3 and CCRF-CEM are large homozygous deletions. A lack of coverage at these positions is expected. For 22 of the 23 remaining variant positions, we obtained sufficient coverage (> 20 fold) to perform reliable variant calling, 21 of these variants are clearly present in the raw sequencing data. One variant was present in the sequencing data but with a coverage of 9 times, precluding reliable variant calling at this position. An overview of mutations and their validation status is available in Table 5. The 2 large homozygously deleted positions in the PTEN gene can be confirmed from the sequencing as well as the qPCR amplification data. Table 6 shows adequate end point fluorescence and Cq values as well as good coverage for the PTEN-4 amplicon comprising the deletion in all but the 2 deleted samples (CCRF-CEM and PC3). Summarizing, the mutation validation rate for this experiment is 23 out of 25 (92%). The only mutation that was missed is designated as complex in the Cosmic database and probably comprises of an inter-chromosomal rearrangement; PCR amplification of the variant allele is impossible with our targeted primer pair. As this variant is heterozygous, the reference allele is amplified, so no deletion is detected by qPCR and the sequencing data only shows the reference allele. Of note is that the 2 deletions in the PTEN gene detected in this dataset are homozygous deletions. A PCR based enrichment technique is probably unable to detect a large heterozygous deletion.

**Table 5:** Mutation validation

**Gene**	**Cel line**	**Position (prot)**	**Position (cdna)**	**Position (genome)**	**QPCR amplification call**	**RAW sequencing data**	**Reference coverage**	**Variant coverage**
APC	HT-29	p.E853*	c.2557G > T	5:112173848	OK	Yes	428	214
APC	HCT-15	p.I1417fs*2	c.4248delC	5:112175539	OK	Yes	2	963
APC	HT-29	p.T1556fs*3	c.4666_4667insA	5:112175957	OK	Yes	608	355
APC	HCT-15	p.R2166*	c.6496C > T	5:112177787	OK	Yes	247	360
BRCA2	HCT-15	p.C1200fs*1	c.3599_3600delGT	13:32912089	OK	Yes	165	114
BRCA2	HCT-15	p.N1784fs*7	c.5351delA	13:32913837	OK	Yes	272	302
MLH1	DU-145	p.?	c.117-2A > T	3:37038108	OK	Yes	0	476
MLH1	IGROV-1	p.S505fs*3	c.1513delA	3:37070378	OK	Yes	5	605
MLH1	CCRF-CEM	p.R100*	c.298C > T	3:37042536	OK	Yes	354	400
MLH1	HCT116	p.S252*	c.755C > A	3:37056000	OK	Yes	0	30
MLH1	CCRF-CEM	p.?	c.790 + 1G > A	3:37056036	OK	Low coverage	3	6
MSH6	IGROV-1	p.F1088fs*2	c.3261delC	2:48030647	OK	Yes	65	650
MSH6	HCT-15	p.D1171fs*4	c.3511_3516 > T	2:48032121	OK	No (complex?)	551	0
MSH6	HCT-15	p.L290fs*1	c.868delC	2:48025990	OK	Yes	214	230
NF2	SN12C	p.?	c.115-1G > C	22:30032739	OK	Yes	0	599
NF2	ACHN	p.R57*	c.169C > T	22:30032794	OK	Yes	2	772
NF2	MDA-MB-231	p.E231*	c.691G > T	22:30057209	OK	Yes	1	817
NOTCH1	CCRF-CEM	p.R1595 > PRLPHNSSFHFLR	c.4783_4784ins36	9:139399362	OK	Yes	96	23
NOTCH1	MOLT-4	p.P2515fs*4	c.7544_7545delCT	9:139390649	OK	Yes	143	155
PTEN	PC3	p.R55fs*1	c.165_1212del1048	10:89685270	Deletion	No coverage	0	0
PTEN	786-O	p.Q149*	c.445C > T	10:89692961	OK	Yes	0	910
PTEN	CCRF-CEM	p.?	c.80_492del413	10:89653782	Deletion	No coverage	0	0
PTEN	MOLT-4	p.K267fs*9	c.800delA	10:89717775.	OK	Yes	34	1499
PTEN	BT-549	p.V275fs*1	c.823delG	10:89720672	OK	Yes	3	159
VHL	786-O	p.G104fs*55	c.311delG	3:10183842	OK	Yes	5	250

Validation rates for all mutations in the NCI60 cell lines included in this publication. The number of reads on the reference and variant bases for each variant position are given in the last 2 columns.

In nonsense or frameshift mutations the * symbol indicates the loss of the amino acid coding potential of the DNA sequence.

**Table 6:** PTEN deletion detection

**Amplicon**	**Chip**	**Sample name**	**End point fluorescence**	**Cq**	**Mean coverage**	**Min coverage**	**Max coverage**	**Coverage stdev**
PTEN-4	chip1	Normal1	3409.9	19.9	766.1	66	1232	314.0
PTEN-4	chip1	Normal1	3219.8	20.1	494.6	40	800	204.5
PTEN-4	chip1	MCF7	3592.8	23.7	450.0	37	720	176.9
PTEN-4	chip1	MCF7	3204.2	23.2	520.2	42	879	216.8
PTEN-4	chip2	786-O	5000.1	22.4	732.0	57	1224	310.2
PTEN-4	chip2	HCT-15	4097.8	19.8	486.7	33	823	201.9
PTEN-4	chip2	MDA-MB-231	4508.4	19.2	386.4	29	643	159.5
PTEN-4	chip2	OVCAR-5	4188.0	19.9	282.8	19	452	107.8
PTEN-4	chip3	MOLT-4	3499.5	19.6	683.9	48	1094	272.9
PTEN-4	chip3	DU-145	4686.3	18.7	364.0	24	610	144.1
PTEN-4	chip3	SN12C	3725.0	19.0	234.9	23	388	90.1
PTEN-4	chip3	BT-549	4036.6	20.0	422.5	45	643	150.1
PTEN-4	chip4	IGROV-1	4000.3	20.0	409.0	35	626	141.1
PTEN-4	chip4	CCRF-CEM	0.0	40.0	0.4	0	2	0.7
PTEN-4	chip4	PC3	0.0	40.0	0.4	0	1	0.5
PTEN-4	chip4	HCT116	3906.9	20.1	627.4	62	1033	246.2
PTEN-4	chip5	Normal1 A	4424.1	20.2	611.7	58	984	245.1
PTEN-4	chip5	ACHN	3996.0	20.2	426.6	28	699	176.5
PTEN-4	chip5	HT-29	4239.3	20.2	256.0	26	396	90.6
PTEN-4	chip5	MCF7	4035.4	20.5	503.0	38	796	196.7
PTEN-4	chip6	Normal1	2955.7	20.5	602.4	39	1015	261.2
PTEN-4	chip6	Normal2	3482.0	20.7	378.8	33	613	145.4
PTEN-4	chip6	Normal2	3440.0	21.1	225.6	18	344	83.2
PTEN-4	chip6	RPMI-8226	3660.0	21.9	365.8	36	571	135.1

Consistent lack of coverage, end point fluorescence and Cq call for the 2 samples with large homozygous deletions in the PTEN gene, spanning the whole of capture assay PTEN-4.

### SNP detection

As a measure of enrichment platform reproducibility and accuracy, the number of correctly called known SNPs is used. The SNP status of the NCI-60 samples is available from the Developmental Therapeutics Program (DTP) website (http://dtp.nci.nih.gov/index.html) as published by [13]. Unfortunately, only 6 SNPs on the Affymetrix 125 K platform fall within the target region of our capture. For these 6 SNPs, the genotype call in the publicly available data was compared to the genotype calls in the 16 NCI-60 samples in our dataset and concurred in 95.6% of the cases. Of the non-concordant SNP calls, 3 occurred in one and the same sample (SN12C) suggesting that a sample naming mix-up might be the cause of this mismatch. We were able to confirm the NF2 mutation in this sample as described by the Cosmic database thus indicating our sample id agreed with that used in the Cosmic database and leading us to doubt the sample id mentioned in the DTP dataset.

A second set of SNP calls is available from an exome sequencing study [14]. We downloaded the variants in the genes included in our target enrichment experiment from the CellMiner tool (http://discover.nci.nih.gov/cellminer/). A total of 473 variants were found in the exome dataset with a genomic position in the target region enriched in our experiment. Of these variants 431 (91.1% with a standard deviation of 5.85% over the 15 cell lines included) were called from our targeted resequencing experiment. From the downloaded data, Abaan et al. do not provide genotype calls but rather variant to reference read ratios. Markedly, the average difference in the variant to reference ratio at each variant position for these two datasets is only half a percentage (0.544%, SD 0.11%) leading us to conclude that genotype calling from our platform is highly congruent with the genotypes called from the exome sequencing dataset if similar algorithms were used in genotype calling.

To increase the number of SNPs in this analysis, we examined the SNP calls for the technical replicates. This analysis not only depends on the accuracy of the capture and the correct co-amplification of both alleles of heterozygous positions, but also on the algorithms used to perform the variant calling. To evaluate the capture platform as objectively as possible (with as little as possible interference of the variant calling algorithm), we looked at the raw variant coverage data for all known polymorphisms (known to dbSNP build 132) in the target region with a coverage of at least 20 and a variant allele called by the genome analysis toolkit in at least one of the technical replicates. For each of these positions, the genotype call was compared in all technical replicates and the raw coverage data was examined (see overview in Table 7 and detailed figures in Additional file 2). For 3 of the samples, technical replicates of the capture and sequencing were performed. These technical replicates deliberately consist of either singleton or duplicate capture PCR reactions on the capture chip to examine if capture efficiency and allelic ratio is depending on the number of PCR replicates in the same chip.

**Table 7:** SNP detection in technical replicates

**Sample**	**Capture reaction**	**PCR replicates**	**Total variants**	**Total variants (union)**	**Variants in comon in between all replicates (intersect)**	**Genotype concordant**	**Genotype discordant**
Normal1 DNA	Duplicate-A	2	145	168 (15 indles)	120 (10 indels)	118 (10 indels)	2
	Duplicate-B	2	133				
	Singelton-A	1	141				
	Singleton-B	1	143				
MCF7	Duplicate-A	2	76	86 (16 indels)	72 (10 indels)	69 (10 indels)	3
	Duplicate-B	2	77				
	Singelton	1	84				
Normal2 DNA	Singelton-A	1	134	149 (14 indels)	121 (11 indels)	119 (11 indels)	2
	Singleton-B	1	133				

The number of consistently and inconsistently called SNP positions across the technical replicates included in this experiment.

Four capture reactions (2 singleton and 2 duplicate) were carried out on the normal1 reference DNA sample. 168 variants were detected in at least one of the replicates, of which 15 indels. 120 (71.4%) of these variants (including 10 indels) were detected in all 4 of the replicates of which only 2 showed a discordance in genotype call (heterozygous vs homozygous) in one of the 4 replicates. Of the 48 variant positions with an inconsistent call in the 4 replicates we closely examined the reason for this discordance from the raw sequencing data. For 5 of the variant positions, the coverage obtained in one or more of the samples was insufficient to perform a variant call. Another five of the 48 variant positions reside in an oligonuleotide repeat (poly N or dinucleotide repeat stretch), traditionally associated with false positive and negative variant calls. For the remaining 38 variant positions, there was a loss or gain of variant information due to changes in the allelic ratio between the reference and variant allele in the replicates. For the MCF7 cell line sample, 3 capture reactions were carried out of which 1 singleton and 2 duplicates PCR captures. For this sample 86 SNP positions (16 indels) were called variant in at least one of the replicates of which 72 (83.7%) (including 10 indels) are common amongst all three replicates, 3 of which showed a discordant genotype call in one of the samples. Again we carefully examined the allele ratio in all 3 replicates of the 14 SNP positions not called consistently in the replicates. Five miscalls could be attributed to genomic repeats at the SNP position. Four could not be called in one or more of the samples due to coverage issues. The remaining 5 were either missed or false positive calls in one of the samples due to a low or borderline coverage for the variant allele in the sample. Excluding the miscalled positions in the repeat regions we can conclude that for the 243 allele calls made for SNPs in the 3 technical replicates, 6 calls (2.4%) could not be made due to coverage issues and 7 (2.9%) appeared dubious due to allelic ratio issues. Performing the same analysis for the normal2 reference DNA sample, we found a total of 149 SNP (14 indel) positions of which 121 (81.2%) (11 indel) were in common between the 2 replicates with only 2 of these not having a concordant genotype. We found 3 SNPs to be in a repeat region, 25 allelic ratio problems and no coverage problems; concluding to allelic ratio issues occurring in 25 out of 292 (total number of SNP calls in both replicates, not in repeat regions) allele calls (8.5%) in these replicates.

Concluding the SNP calling data on the technical replicates, we have a total of 1228 SNP positions (the sum of the number of SNP positions per sample across the replicates times the number of replicates for that sample) in all the technical replicates. Only 14 SNP calls (1.1%) could not be made due to coverage issues (this is independent from the capture being performed by single or duplicate PCR reactions). 9.2% of the SNP calls could not be made due to a difference in the allelic ratio (for ease of counting we assume the SNP call not being made due to an allelic loss as opposed to a false positive call at the variant position having occurred). Upon separate analysis of singleton vs duplicate PCR replicate capture reactions, we observed a slightly higher (non-significant) number of these types of errors occurring in the singleton, namely 8.1% or 33 out of 305 of the SNPs, versus 5.2% or 21 of 232 of the SNPs for the duplicate PCR reaction captures (Chi squared test p > 0.05). We can conclude that only a minor fraction of SNP positions could not be called due to coverage issues and there is no influence on SNP calling based on the number of PCR enrichment reactions. As we have no arguments to state that the enrichment should be performed in duplicate, we conclude that the nanowell PCR capture is highly reproducible and reliable for singleton PCR reactions. We do see some issues with technical reproducibility of allele calling on our platform due to variable allelic ratio of heterozygous SNP positions. Unfortunately, we cannot compare our data with that of other capture platforms as no detailed analysis like this has been published for any of these platforms.

## Conclusions

Today, three different sequence enrichment methodologies are available [15]: the PCR based (Access Array by Fluidigm, Directseq by Raindance Technologies and Ampliseq by Life Technologies), hybridization based (Sureselect by Agilent and Nimblegen by Roche) and hybridization-extension based (Haloplex by Agilent and Truseq by Illumina) methods. Each of these technologies has its strengths and limitations. PCR based methods are generally accepted to have the best overall sensitivity and specificity but are limited in target size mainly due to the primer cost [16]. The PCR based platforms thus are targeted towards the diagnostic resequencing market where primers can be reused. The pure hybridization based approaches are unable to capture some types of regions, mainly due to probe design issues around genomic repeats and pseudogenes, but scale easily and thus are currently geared towards whole exome sequencing applications [15].

The hybridization-extension based technology from Agilent (Haloplex) and Illumina (Truseq) are both available for small targeted resequencing experiments as well as for whole exome resequencing. At the time of writing, experience with these novel platforms is limited so no solid data on their real life performance is available in the literature.

In this study we demonstrate a novel quantitative PCR based sequence capture platform that has distinctive advantages over the currently existing capture platforms. With up to 5000 reaction wells per chip and the possibility to efficiently amplify amplicons with a large range in length, the maximum capture target size is similar to both the Ampliseq and Raindance platforms and is surpassing the Fluidigm array based platform that is limited to 96 individual assays. However, similar to the Fluidim platform and in contrast to the Ampliseq and Raindance platforms, the new platform also offers a large flexibility in experimental design. In contrast to capture reactions that are carried out in a single invariable multiplexed reaction, the chip based amplification allows a flexible combination of samples and amplicons without interference. Other similar chip based platforms have already been described and have proven their reliability [17,18]. But none have ever been used to perform sequence capture.

There is only scarce amount of literature available comparing the performance statistics of the above referenced platforms. Jones et al. [19] report a targeted resequencing of 24 genes involved in congenital disorders of glycosilation in 12 positive control patient samples on both the Raindance and Fluidigm platform. A perfect mutation detection rate is reported for both. A direct comparison of the performance of these platforms with our dataset is not possible due to the differences in target region, sequencing and analysis strategy. The reported exon failure rates (low or no coverage) of 15 out of 225 (6.9%) for Raindance and 13 out of 215 (6.0%) for Fluidigm are quite similar to the failure rate observed in our experiments. The Fluidigm platform is also extensively evaluated in a study of mutation discovery in patients with nephronophthisis-associated ciliopathy (11 genes in 192 patients) [20]. The authors report a mutation validation rate of 90% and a 93.2% exon capture success rate as defined by a coverage > 30 fold. Other real life but smaller gene set coverage statistics for the Fluidigm platform are reported by Hollants et al. [21]; 37 of 38 (97%) amplicons captured successfully and Schlipf et al. [22] with 15 of 17 (88%) amplicons captured successfully. None of the above referenced studies evaluate the coverage uniformity as a metric. The Raindance capture platform is evaluated in several studies. Hu et al. have selectively resequenced 86 genes implicated in X linked mental retardation [23]. They report an 91% amplicon design success rate and an average of 88.5% of the target bases receiving adequate coverage across the samples. 90% of the bases have a coverage of at least 29% of the mean coverage. The reproducibility of the capture is indicated by the reported 0.84 and 0.90 average pairwise correlation for the per base and per amplicon coverage rates respectively across all the samples. In their whole chromosome X exome resequencing experiment Mondal et al. report an amplicon design success rate of 98% and a subsequent capture success rate of 97.3% described as the percentage of the target bases covered by at least one read [24].

In a diagnostic resequencing effort for congenital deafness genes, carried out with the Raindance platform, the authors indicate a primer design success rate for 99.9% of the target bases and 95% of these bases reaching adequate coverage [25]. A diagnostic test for congenital muscular dystrophy including some genes with high GC nucleotide content is reporting 84 to 95% capture success on the target region for the samples included in the analysis [26]. Highly consistent in all these Raindance capture based publications is a large number of off-target or unmappable reads ranging between 40 and 70% of the total reads indicating some issues with off-target amplification and the downstream library preparation or data analysis procedure [23-26].

Literature on the capture success rate of both the Ampliseq and Haloplex platform is, due to the novelty of the platforms, limited. No publications exist elaborating on the performance characteristics of the platforms. The underlying technology for the Haloplex platform is described in a publication by Johansson et al. who mention a high capture success rate of over 98% and an inter sample coverage correlation of 0.98 on a limited set of samples in controlled circumstances [27].

The performance characteristics of our platform are similar to, if not outperforming some of the best statistics currently published on targeted resequencing. Considering that our PCR assays were not optimized, the 93.6 percentage reproducible assay enrichment success rate can be considered high compared to competing platforms. By performing some assay optimization, assay replacement, or by including multiple assays for the same genomic target, our capture success rate can be increased, which is important for diagnostic applications. One major advantage over competing platforms is the flexibility in the assay dispensing in the capture chips; we can easily exchange badly performing assays with new designs, or include additional targets of interest. The use of individual qPCR reactions clearly resulted in a very high coverage uniformity. This together with the high amplification specificity and a high degree of successful read mappings results in a highly efficient capture platform reducing the amount of over sequencing needed to achieve adequate coverage. A potential downside of these individual PCR reactions can be the amount of DNA required to perform a capture reaction on a sample which scales linearly with the number of targets a user wants to capture. Although the amount of input DNA required for a single reaction is low (360 pg) the capture of the maximum number of 5184 targets on this platform is roughly 1800 ng which might not be available for all diagnostic samples. However, one may consider to introduce a sample pre-amplification step, as successfully done in Sjöblom et al. [28].

The high mutation and SNP calling validation rates show the potential of this platform to be integrated into diagnostic workflows. Based on the platform we describe here, WaferGen Biosystems meanwhile has developed a low-cost target enrichment platform consisting of a single-sample nanodispenser and a PCR system that is able to run 2 chips at the same time. This platform is compatible with different types of chips containing between 1296 and 5184 PCR reactions, making it possible to run more than 50,000 single PCR capture reactions per day. The discordance in the SNP calling for the technical replicates warrants a note of caution in applying captured resequencing platforms in routine diagnostics without proper validation. No adequate sensitivity or specificity assessment for any next generation capture and sequencing platform currently exist and thus we have no means of comparing our statistics to the ones of competing platforms. We would like to urge other researchers to include technical replicates in their evaluation of any next generation sequencing platform, especially when aiming to design workflows with potential diagnostic applications.

## Abbreviations

qPCR: Quantitative polymerase chain reaction; Cq: Quantitation cycle; SNP: Single nucleotide polymorphism.

## Competing interests

SD, WD, JD and SH are employees of WaferGen Biosystems that has launched a target enrichment platform as a commercial product, in part based on the results of this study.

## Authors’ contributions

BDW optimized the target enrichment platform, performed the sequencing data analysis, the statistical analysis and drafted the manuscript. SD carried out the target enrichments. SL and JH designed the PCR assays. SD, WD, JD and SH were involved in optimizing the Smart Chip platform for target enrichment. JV and SD conceived of the study, and participated in its design and coordination. All authors read and approved the final manuscript.

## Supplementary Material

Additional file 1 **Primer information.** Excel file containing the amplicon positions and sequence. Click here for file

Additional file 2 **Technical replicate SNP failures.** Excel file with a list of inconsistently called SNPs in the technical replicates. The reason for the inconsistency as well as the allelic ratio per replicate are given. Click here for file

## Supporting data

The data set supporting the results of this article is available in the EMBL repository, PRJEB4408 http://www.ebi.ac.uk/ena/data/view/PRJEB4408.

## References

[B1] Mardis ER The impact of next-generation sequencing technology on genetics Trends Genet 2008 24 133 141 10.1016/j.tig.2007.12.007 18262675 PMC2680276

[B2] Mardis E Ding L Dooling D Larson D McLellan M Chen K Koboldt D Fulton R Delehaunty K McGrath S Fulton L Locke D Magrini V Abbott R Vickery T Reed J Robinson J Wylie T Smith S Carmichael L Eldred J Harris C Walker J Peck J Du F Dukes A Sanderson G Brummett A Clark E McMichael J Recurring mutations found by sequencing an acute myeloid leukemia genome N Engl J Med 2009 361 1058 1066 10.1056/NEJMoa0903840 19657110 PMC3201812

[B3] Pleasance ED Keira Cheetham R Stephens PJ Mcbride DJ Humphray SJ Greenman CD Varela I Lin M-L Ordóñez GR Bignell GR Ye K Alipaz J Bauer MJ Beare D Butler A Carter RJ Chen L Cox AJ Edkins S Kokko-Gonzales PI Gormley NA Grocock RJ Haudenschild CD Hims MM James T Jia M Kingsbury Z Leroy C Marshall J Menzies A A comprehensive catalogue of somatic mutations from a human cancer genome Nature 2010 463 191 196 10.1038/nature08658 20016485 PMC3145108

[B4] Pleasance ED Stephens PJ O'meara S Mcbride DJ Meynert A Jones D Lin M-L Beare D Lau KW Greenman C Varela I Nik-Zainal S Davies HR Ordoñez GR Mudie LJ Latimer C Edkins S Stebbings L Chen L Jia M Leroy C Marshall J Menzies A Butler A Teague JW Mangion J Sun YA McLaughlin SF Peckham HE Tsung EF A small-cell lung cancer genome with complex signatures of tobacco exposure Nature 2010 463 184 190 10.1038/nature08629 20016488 PMC2880489

[B5] Bell D Berchuck A Birrer M Chien J Cramer DW Dao F Dhir R DiSaia P Gabra H Glenn P Godwin AK Gross J Hartmann L Huang M Huntsman DG Iacocca M Imielinski M Kalloger S Karlan BY Levine DA Mills GB Morrison C Mutch D Olvera N Orsulic S Park K Petrelli N Rabeno B Rader JS Sikic BI Integrated genomic analyses of ovarian carcinoma Nature 2011 474 609 615 10.1038/nature10166 21720365 PMC3163504

[B6] Larsen LA Christiansen M Vuust J Andersen PS High throughput mutation screening by automated capillary electrophoresis Comb Chem High Throughput Screen 2000 3 393 409 10.2174/1386207003331508 11032956

[B7] Mitchelson KR The use of capillary electrophoresis for DNA polymorphism analysis Mol Biotechnol 2003 24 41 68 10.1385/MB:24:1:41 12721495

[B8] Shoemaker RH The NCI60 human tumour cell line anticancer drug screen Nat Genet 2006 6 813 823 10.1038/nri1943 16990858

[B9] Forbes SA Bindal N Bamford S Cole C Kok CY Beare D Jia M Shepherd R Leung K Menzies A Teague JW Campbell PJ Stratton MR Futreal PA COSMIC: mining complete cancer genomes in the catalogue of somatic mutations in cancer Nucleic Acids Res 2010 39 D945 D950 Database 20952405 10.1093/nar/gkq929 PMC3013785

[B10] Li H Durbin R Fast and accurate short read alignment with burrows-wheeler transform Bioinformatics 2009 25 1754 1760 10.1093/bioinformatics/btp324 19451168 PMC2705234

[B11] McKenna A Hanna M Banks E Sivachenko A Cibulskis K Kernytsky A Garimella K Altshuler D Gabriel S Daly M DePristo MA The genome analysis toolkit: a MapReduce framework for analyzing next-generation DNA sequencing data Genome Res 2010 20 1297 1303 10.1101/gr.107524.110 20644199 PMC2928508

[B12] Ikediobi ON Davies H Bignell G Edkins S Stevens C O'Meara S Santarius T Avis T Barthorpe S Brackenbury L Buck G Butler A Clements J Cole J Dicks E Forbes S Gray K Halliday K Harrison R Hills K Hinton J Hunter C Jenkinson A Jones D Kosmidou V Lugg R Menzies A Mironenko T Parker A Perry J Mutation analysis of 24 known cancer genes in the NCI-60 cell line set Mol Cancer Ther 2006 5 2606 2612 10.1158/1535-7163.MCT-06-0433 17088437 PMC2705832

[B13] Garraway LA Widlund HR Rubin MA Getz G Berger AJ Ramaswamy S Beroukhim R Milner DA Granter SR Du J Lee C Wagner SN Li C Golub TR Rimm DL Meyerson ML Fisher DE Sellers WR Integrative genomic analyses identify MITF as a lineage survival oncogene amplified in malignant melanoma Nature 2005 436 117 122 10.1038/nature03664 16001072

[B14] Abaan OD Polley EC Davis SR Zhu YJ Bilke S Walker RL Pineda M Gindin Y Jiang Y Reinhold WC Holbeck SL Simon RM Doroshow JH Pommier Y Meltzer PS The exomes of the NCI-60 panel: a genomic resource for cancer biology and systems pharmacology Cancer Res 2013 73 4372 4382 10.1158/0008-5472.CAN-12-3342 23856246 PMC4893961

[B15] Mertes F Elsharawy A Sauer S van Helvoort JMLM van der Zaag PJ Franke A Nilsson M Lehrach H Brookes AJ Targeted enrichment of genomic DNA regions for next-generation sequencing Bfgoxfordjournalsorg 2011 10 374 386 10.1093/bfgp/elr033 PMC3245553 22121152

[B16] Mamanova L Coffey AJ Scott CE Kozarewa I Turner EH Kumar A Howard E Shendure J Turner DJ Target-enrichment strategies for next-generation sequencing Nat Meth 2010 7 111 118 10.1038/nmeth.1419 20111037

[B17] Dahl A Sultan M Jung A Schwartz R Lange M Steinwand M Livak KJ Lehrach H Nyarsik L Quantitative PCR based expression analysis on a nanoliter scale using polymer nano-well chips Biomed Microdevices 2007 9 307 314 10.1007/s10544-006-9034-2 17203381

[B18] Mertes F Biens K Lehrach H Wagner M Dahl A High-throughput universal probe salmonella serotyping (UPSS) by nanoPCR J Microbiol Methods 2010 83 217 223 10.1016/j.mimet.2010.09.005 20869995

[B19] Jones MA Bhide S Chin E Ng BG Rhodenizer D Zhang VW Sun JJ Tanner A Freeze HH Hegde MR Targeted polymerase chain reaction-based enrichment and next generation sequencing for diagnostic testing of congenital disorders of glycosylation Genet Med 2011 13 921 932 10.1097/GIM.0b013e318226fbf2 21811164 PMC3398737

[B20] Halbritter J Diaz K Chaki M Porath JD Tarrier B Fu C Innis JL Allen SJ Lyons RH Stefanidis CJ Omran H Soliman NA Otto EA High-throughput mutation analysis in patients with a nephronophthisis-associated ciliopathy applying multiplexed barcoded array-based PCR amplification and next-generation sequencing J Med Genet 2012 49 756 767 10.1136/jmedgenet-2012-100973 23188109

[B21] Hollants S Redeker EJW Matthijs G Microfluidic amplification as a tool for massive parallel sequencing of the familial hypercholesterolemia genes Clin Chem 2012 58 717 724 10.1373/clinchem.2011.173963 22294733

[B22] Schlipf NA Schüle R Klimpe S Karle KN Synofzik M Schicks J Riess O Schöls L Bauer P Amplicon-based high-throughput pooled sequencing identifies mutations in CYP7B1 and SPG7 in sporadic spastic paraplegia patients Clin Genet 2011 80 148 160 10.1111/j.1399-0004.2011.01715.x 21623769

[B23] Hu H Wrogemann K Kalscheuer V Tzschach A Richard H Haas SA Menzel C Bienek M Froyen G Raynaud M Van Bokhoven H Chelly J Ropers H Chen W Mutation screening in 86 known X-linked mental retardation genes by droplet-based multiplex PCR and massive parallel sequencing HUGO J 2010 3 41 49 21836662 10.1007/s11568-010-9137-y PMC2882650

[B24] Mondal K Shetty AC Patel V Cutler DJ Zwick ME Targeted sequencing of the human X chromosome exome Genomics 2011 98 260 265 10.1016/j.ygeno.2011.04.004 21524701 PMC3154473

[B25] Schrauwen I Sommen M Corneveaux JJ Reiman RA Hackett NJ Claes C Claes K Bitner-Glindzicz M Coucke P Van Camp G Huentelman MJ A sensitive and specific diagnostic test for hearing loss using a microdroplet PCR-based approach and next generation sequencing Am J Med Genet 2012 161 145 152 23208854 10.1002/ajmg.a.35737

[B26] Valencia CA Ankala A Rhodenizer D Bhide S Littlejohn MR Keong LM Rutkowski A Sparks S Bonnemann C Hegde M Comprehensive mutation analysis for congenital muscular dystrophy: a clinical PCR-based enrichment and next-generation sequencing panel PLoS ONE 2013 8 e53083 10.1371/journal.pone.0053083 23326386 PMC3543442

[B27] Johansson H Isaksson M Sorqvist EF Roos F Stenberg J Sjoblom T Botling J Micke P Edlund K Fredriksson S Kultima HG Ericsson O Nilsson M Targeted resequencing of candidate genes using selector probes Nucleic Acids Res 2011 39 e8 e8 10.1093/nar/gkq1005 21059679 PMC3025563

[B28] Sjoblom T Jones S Wood LD Parsons DW Lin J Barber TD Mandelker D Leary RJ Ptak J Silliman N Szabo S Buckhaults P Farrell C Meeh P Markowitz SD Willis J Dawson D Willson JKV Gazdar AF Hartigan J Wu L Liu C Parmigiani G Park BH Bachman KE Papadopoulos N Vogelstein B Kinzler KW Velculescu VE The consensus coding sequences of human breast and colorectal cancers Science 2006 314 268 274 10.1126/science.1133427 16959974

